# 2304. Surveillance strategies for the detection of new SARS-CoV-2 variants across epidemiological contexts

**DOI:** 10.1093/ofid/ofad500.1926

**Published:** 2023-11-27

**Authors:** Kirstin Oliveira Roster, Stephen M Kissler, Enoma Omoregie, Jade Wang, Helly Amin, Steve Di Lonardo, Scott Hughes, Yonatan H Grad

**Affiliations:** Harvard School of Public Health, Boston, Massachusetts; University of Colorado Boulder, Boston, Massachusetts; NYC Department of Health and Mental Hygiene, New York City, New York; NYC Department of Health and Mental Hygiene, New York City, New York; NYC Department of Health and Mental Hygiene, New York City, New York; NYC Department of Health and Mental Hygiene, New York City, New York; NYC Department of Health and Mental Hygiene, New York City, New York; Harvard Chan School of Public Health, Boston, Massachusetts

## Abstract

**Background:**

Rapid identification of new SARS-CoV-2 variants is a critical component of the public health response to the COVID-19 pandemic. However, we lack a quantitative framework to assess the expected performance of sampling strategies in varying epidemic contexts.

**Methods:**

To address this gap, we used a multi-patch stochastic model of SARS-CoV-2 spread in New York City to evaluate the impact of the volume of testing and sequencing, geographic representativeness of sampling, location and timing of variant emergence, and relative variant transmissibility on the time to first detection of a new variant.

**Results:**

The strategy of targeted sampling of likely emergence locations offered the most improvement in detection speed. Increasing sequencing capacity reduced detection time more than increasing testing volumes. The relative transmissibility of the new variant and the epidemic context of variant emergence also influenced detection times, showing that individual surveillance strategies can result in a wide range of detection outcomes, depending on the underlying dynamics of the circulating variants.

Detection time by test volume and fixed sequencing capacity.

**Figure:**
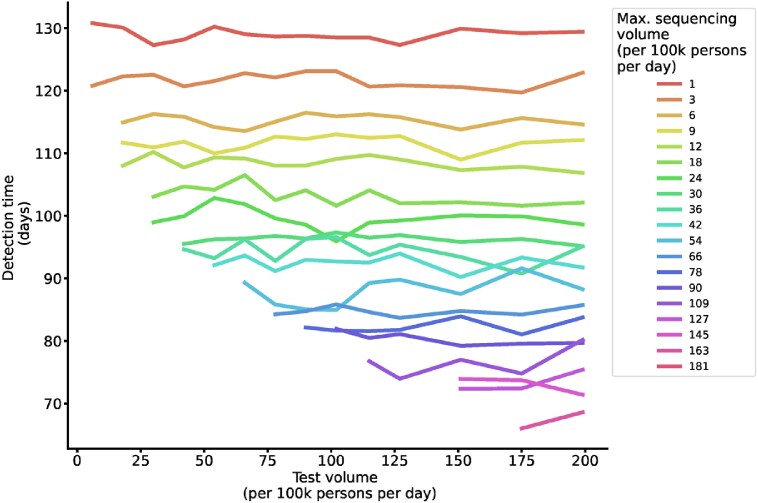

Lines depict the mean duration between variant introduction and detection in days as a function of daily testing volume, colored by the maximum sequencing volume (A), and as a function of daily maximum sequencing volume, colored by the test volume (B) (at variant introduction 50 days after the prior variant and baseline sampling strategy).

Detection time by proportion of tests allocated in a single location.

**Figure:**
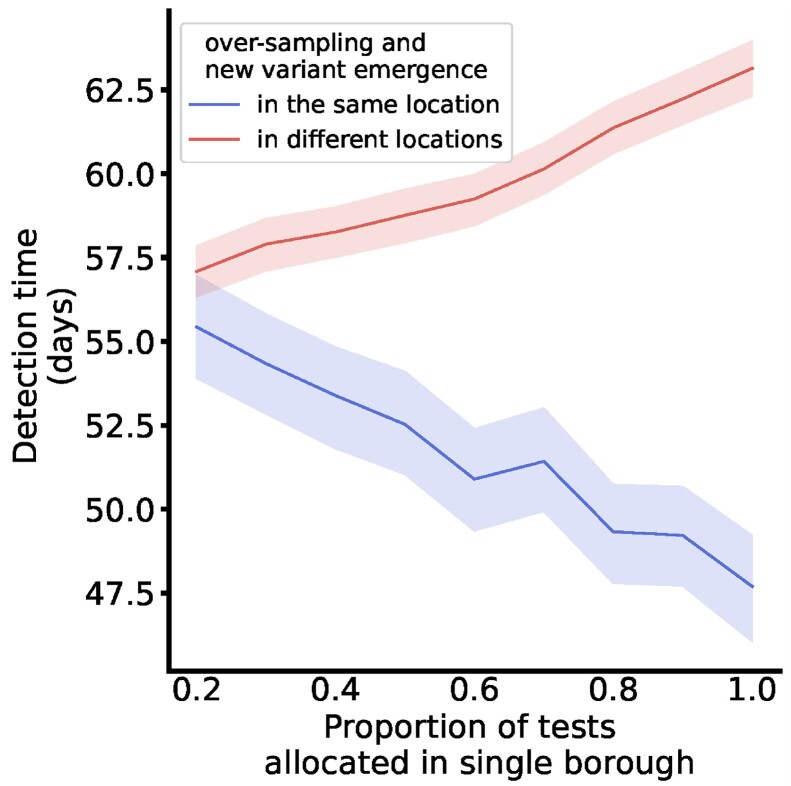

Lines depict the average detection time for scenarios where between 20% and 100% of tests are sampled from a single location, and the remaining tests are evenly distributed across the remaining locations by population size. The lines distinguish between scenarios where the variant emerged in the primary allocation location, i.e., test over-sampling and emergence occurred in the same location (blue), and scenarios where the variant emerged in one of the other locations, i.e., test over-sampling and emergence occurred in different locations (red). Ribbons depict the 95% confidence interval for the detection time.

**Conclusion:**

These findings help contextualize the design, interpretation, and trade-offs of genomic surveillance strategies.

**Disclosures:**

**Stephen M. Kissler, PhD**, ModernaTx: Advisor/Consultant **Yonatan H. Grad, MD, PhD**, Day Zero Diagnostics: Board Member|GSK: Advisor/Consultant

